# Cytotoxicity and genotoxicity of Bio-C Repair, Endosequence BC Root Repair, MTA Angelus and MTA Repair HP

**DOI:** 10.1590/0103-6440202305047

**Published:** 2023-05-15

**Authors:** Suene Moçato Siguematsu Abrão, Danielle Gregorio, Monalisa Kethleen Costa De Azevedo, Graziela Garrido Mori, Regina Célia Poli-Frederico, Luciana Prado Maia

**Affiliations:** 1 Graduate Program in Dentistry of the University Pitagoras Unopar (UNOPAR), Londrina, Paraná, Brazil.; 2 Graduate Program in Dentistry of the University of Western Sao Paulo (UNOESTE). Presidente Prudente, São Paulo, Brazil.

**Keywords:** osteoblasts, calcium silicate, mineral trioxide aggregate, genotoxicity, cytotoxicity

## Abstract

The aim was to evaluate *in vitro* cytotoxicity and genotoxicity of Bio-C Repair (BCR), compared to Endosequence BC Root Repair (ERRM), MTA Angelus (MTA-Ang), and MTA Repair HP (MTA-HP). MC3T3 osteoblastic cells were exposed to extracts of the repairing bioceramic cements. After 1, 3, and 7 days, cytotoxicity and genotoxicity were evaluated by MTT and Micronucleus tests, respectively. Cells not exposed to biomaterials were used as a negative control. Data were compared using ANOVA two-way, followed by the Tukey Test (α=5%). MTA-Ang and MTA-HP showed no difference in relation to control regarding cytotoxicity in any experimental times. BCR and ERRM reduced cell viability after 3 and 7 days (*p*<0.05); however, the reduction caused by BCR was less than that caused by ERRM. Considering the micronucleus formation, all biomaterials caused an increase after 3 and 7 days (*p*<0.05), being greater for the BCR and ERRM groups**.** It can be concluded that BCR is non-cytotoxic in osteoblastic cells, as well as MTA-Ang e MTA Repair HP. BCR and ERRM showed greater genotoxicity than others tested biomaterials.

## Resumo

O objetivo foi avaliar in vitro a citotoxicidade e genotoxicidade do Bio-C Repair (BCR), em comparação com o Endosequence BC Root Repair (ERRM), MTA Angelus (MTA-Ang) e MTA Repair HP (MTA-HP). As células osteoblásticas MC3T3 foram expostas aos extratos dos cimentos biocerâmicos reparadores. Após 1, 3 e 7 dias, a citotoxicidade e a genotoxicidade foram avaliadas pelos testes MTT e Micronúcleo, respectivamente. Células não expostas aos biomateriais foram utilizadas como controle negativo. Os dados foram comparados por ANOVA de dois fatores, seguido do Teste de Tukey (*p* = 5 %). MTA-Ang e MTA-HP não apresentaram diferença em relação ao controle quanto à citotoxicidade em nenhum dos tempos experimentais. BCR e ERRM reduziram a viabilidade celular após 3 e 7 dias (*p* < 0,05); no entanto, a redução causada pelo BCR foi menor que aquela causada pelo ERRM. Todos os biomateriais causaram aumento na formação de micronúcleos após 3 e 7 dias (*p* < 0,05), sendo maior para os grupos BCR e ERRM. O BCR não é citotóxico em células osteoblásticas, assim como cimentos MTA-Ang e MTA Repair HP. BCR e ERRM apresentaram maior genotoxicidade do que outros biomateriais testados.

## Introduction

Mineral trioxide aggregate (MTA) is considered a classic bioceramic cement, bioactive, biocompatible, osteoinductive, and antibacterial, with excellent sealing power, dimensional stability, hardness, and slight expansion ^1^. The cellular response to MTA is already well established in the literature and many *in vitro* and *in vivo* studies that evaluated cellular and tissue interactions with MTA have demonstrated its biocompatibility ^2^ ^,^ ^3^ ^,^ ^4^ ^,^ ^5^ ^,^ ^6^ ^,^ ^7^ ^,^ ^8^ ^,^ ^9^. It has several applications in dentistry, such as root perforation, internal or external resorption, retrofilling in apical microsurgery, pulp capping, apexification, apexogenesis, and pulpotomy ^2^. Despite this, MTA has some negative characteristics, such as dentin staining ^4^; the requirement of mixing, which can lead to waste and technical sensitivity; poor dispersion and high porosity; difficult to mix due to its sandy consistency; it needs specific instruments and takes a long time to set ^5^ ^,^ ^10^ ^,^ ^11^.

In order to overcome these deficiencies, new versions of this biomaterial were developed from changes in original formulation or composition, with the aid of nanotechnology maintaining the biological benefits and increasing mechanical resistance ^12^ ^,^ ^13^. In this context, in 2016, the MTA Repair HP (MTA-HP; Angelus, Londrina, Brazil) was introduced, which has the differential of replacing the bismuth oxide as radiopacifier for calcium tungstate, which does not cause staining of the root or dental crown, and the replacement of distilled water with a liquid containing water plus an organic plasticizer, which makes the cement more plastic, facilitating handling and insertion ^14^.

With the advent of nanotechnology, pre-mixed bioceramics emerged, and they have hydrophilic characteristics that allow their use in operating environments with blood contamination, resulting in less technical sensitivity ^6^. One such example is the Endosequence® BC RRM ™ (ERRM; Brasseler, Savannah, GA), available in the form of a condensable mass or paste packaged in a syringe, with uniform consistency, which makes application and handling easy. Additionally, in 2019, the Bio-C Repair (BCR; Angelus, Londrina, Brazil), another premixed reparative bioceramic cement, presented in the form of paste, packaged in a threadable syringe, was commercially available ^14^.

To improve properties, many dental materials can be reformulated and further investigations are needed to ensure their security for clinical use ^15^ ^,^ ^16^. Hence, analyses to determine the biological characteristics of biomaterials are crucial since they maintain contact with different cells and tissue, specifically dental pulp, periodontal ligament, and bone ^17^. Among those characteristics, the absence of harmful effects and genetics consequences must be considered, explaining the benefit of the cytotoxicity and genotoxicity assays as initial analyses to indicate the use of new biomaterials ^18^ ^,^ ^19^. Contemplating on these parameters, the present study is important to certify the clinical application of BCR, a reformulated material ^14^.

BCR is a reformulated material ^14^ and, to our knowledge, only two studies ^20^ ^,^ ^21^ investigated its biological properties, comparing it with bioceramic cements of different generations. Because of that and considering the importance of biological properties, his study was proposed. Thus, this study aimed to assess the cytotoxicity and genotoxicity of BCR, compared to Endosequence BC Root Repair Material, MTA Angelus (Angelus) and MTA-HP. The null hypothesis was there would be no significant differences between BCR and the other cements tested.

## Materials and methods

### Odontoblast-like cell cultures

Mouse pre-osteoblastic cell line MC3T3-E1 subclone 14 - RRID:CVCL_0409 (American Type Culture Collection, VA) were cultivated in alpha-minimum essential medium (Alfa-MEM; Gibco/Thermo Fisher Scientific, Waltham, MA, USA), 10 % fetal bovine serum (FBS; Gibco/Thermo Fisher Scientific), 1% antibiotic-antimycotic (Gibco/Thermo Fisher Scientific). The cells were maintained in a humidified environment at 37°C with 5% CO_2_ and 95% atmospheric air. At subconfluence cells were subcultured and plated in 96-well polystyrene plates (Corning Inc., Corning, NY, USA) at cell density of 10 000 cells/well with osteogenic medium, composed of expansion medium supplemented with 7 mM β-glycerophosphate (Sigma-Aldrich, St. Louis, MO, USA) and 50 μg/mL ascorbic acid (Sigma-Aldrich) for 24 h before exposure to the extracts of the cements.

### Cements manipulation and culture exposure

The cements used in this experiment were Bio-C Repair (BCR), Endosequence BC repair (ERRM), MTA Angelus (MTA-Ang) and MTA Repair HP (MTA-HP), and additional information of these materials are presented in Box 1. All materials were handled according to the manufacturer’s instructions to prepare samples cylindrically shaped, with 2x4 mm under sterile conditions. Immediately after manipulation, the samples were individually covered with sterile cotton wool moistened with distilled water, as previously recommended ^5^ ^,^ ^10^ ^,^ ^11^ ^,^ ^22^ and incubated for 7 days ^5^ ^,^ ^11^ in a humidified environment at 37°C with 5% CO_2_ and 95% atmospheric air with no cotton changes. Then, osteogenic medium was exposed during 24 h to experimental samples. Osteogenic medium was used as control group (Group C).

**Box 1:**
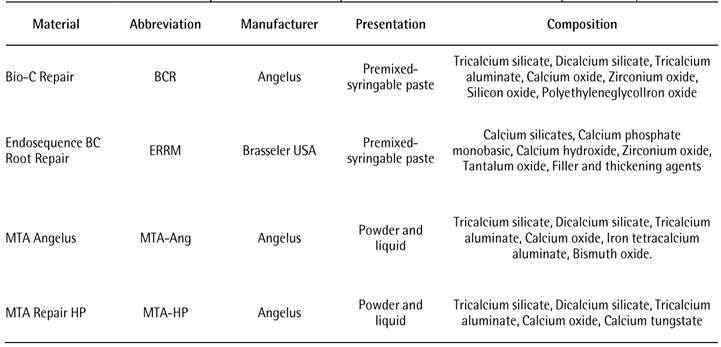
Materials, manufacturer, presentation and composition of the materials used in the present study.

### Cytotoxicity test

Based on the standards set by ISO 10993-5:2009 ^3^ ^,^ ^23^, cytotoxicity was evaluated at 1, 3, and 7 days by the mitochondrial tetrazolium test (MTT; Sigma-Aldrich, St. Louis, MO, USA) ^24^. Briefly, aliquots of MTT at 5 mg/ml in phosphate-buffered saline solution (PBS; Gibco) were prepared and the cells were then incubated with 10% solution in culture media for 4 hours at 37ºC, in a humidified atmosphere containing 5% CO_2_. After this period, the entire solution was removed and 100 µL of dimethyl sulfoxide solution (DMSO) was added to each well under stirring for 5 minutes for complete solubilization of the precipitate formed. The colorimetric measurement was performed on a spectrophotometer (μQuantTM; BioTek, Winooski, VT, USA) using a wavelength of 570 nm. The results were expressed as a relative percentage of the negative control, using the following formula: ((OD_T_-OD_B_)x100/(OD_C_-OD_B_)), where OD = optical density, T = treatment; B = blank; C = control. The material that induced a reduction in cell viability greater than 30% was considered cytotoxic ^21^.

### Genotoxicity Test

Genotoxicity was evaluated at days 1, 3 and 7 by the micronucleus (Mn) test ^23^ ^,^ ^25^. Briefly, the wells were washed with phosphate buffered solution (PBS) 3 times, cells were fixed with 10% neutral formaldehyde for 10 minutes and washed again with PBS. After that, 200 μL of PBS and a drop of FluoroShield with DAPI (Merck) were added to each well and the plates were shaken for 5 minutes, under light protection. The wells were analyzed with a fluorescence microscope (Eclipse Ti Nikon - UV-2ª filter, wavelength of 330-380/420-700 nm of absorption/emission) by counting the number of micronuclei present in 1000 cells/well ^25^. The criteria established by Fenech ^25^ was used to identify and standardize the Mn count.

**Figure 1:**
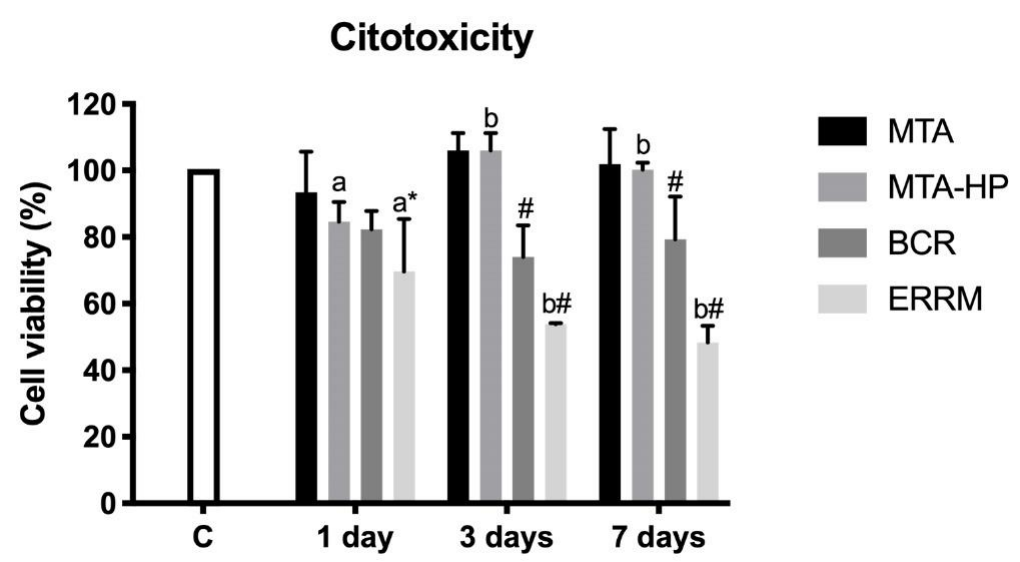
Cell viability of all experimental groups over the experimental period. Bars indicate mean ± standard deviation of the percentage in relation to the control group. Different letters indicate a significant difference in intra-group comparisons; * = significant difference compared to groups C and MTA-Ang; # = significant difference compared to all other groups

### Statistical analysis

Data distribution was analyzed using the Shapiro-Wilk test. Two-way ANOVA was used for intra and group comparisons, followed by the Tukey test for multiple comparisons, considering a 5% significance level. GraphPad Prism 7 software (GraphPad Software, California, USA) was used for all analyses and to create the graphics.

## Results

### Cytotoxicity

**Figure 2:**
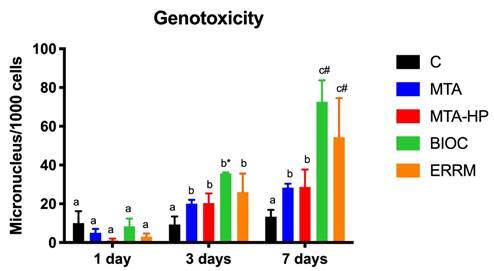
Number of changes consistent with genotoxicity in the different groups after 1, 3 and 7 days of treatment. The bars indicate mean ± standard deviation of the number of micronucleus/1000 cells. Different letters indicate a significant difference in intra-group comparisons; * = significant difference compared to the BCR group; # = significant difference compared to all other groups.

At all experimental periods, MTA-Ang and MTA-HP did not cause a significant change in cell viability compared to group C. BCR reduced cell viability after 3 and 7 days of treatment compared to group C (*p* < 0.022), but this reduction was less than 30%, not being considered a cytotoxic material. ERRM led to an even greater reduction in cell viability, showing a significant difference in relation to the control group after 1 day (*p* < 0.001), and in relation to all other groups at 3 (*p* <0.026) and 7 days (*p* <0.020), presenting potential toxicity at all experimental times (Figure 2). In the intragroup analysis, a difference in cell viability was observed over time only for MTA-HP and ERRM, with MTA-HP significantly increasing cell viability from 1 to 3 days (*p* = 0.005), while the ERRM led to a significant decrease in the same period (p = 0.038) (Figure 1)

### Genotoxicity

On the first day of treatment, all materials evaluated showed a number of micronuclei similar to group C. However, after 3 days, BCR led to a significant increase when compared to group C (*p* = 0.001). At 7 days, both BCR and ERRM showed significantly higher values than groups C, MTA-Ang and MTA-HP (*p* <0.001) (Figure 3). In the intra-group analysis, it was observed that MTA-Ang and MTA-HP led to an increase from 1 to 3 days, with no difference between 3 and 7 days (*p* = 0.277); while BCR and ERRM continued to significantly increase the number of Mn from 3 to 7 days (*p* <0.008) (Figure 2).

## Discussion

In the present study, while MTA-Ang and MTA-HP do not present cytotoxicity or genotoxicity in osteoblastic cells, BCR and ERRM, in spite of non-cytotoxicity, had greater genotoxicity than conventional MTA in the long term. These results reject the null hypothesis that it was no significant differences between BCR and the other types of cement.

Analyses considering cell viability and genotoxicity are important to assess cell damage and the biological effect of new biomaterials ^10^, justifying the tests used in this study. The semi-quantitative cytotoxicity assay using the MTT method allows for measuring the percentual of viable cells, and what possibility of comparing the viability between the groups ^24^. This result is obtained after the reduction of the yellow MTT salt by the mitochondrial activity of viable cells into blue/purple-colored formazan crystals, which can be dissolved in an organic solvent, which concentration can be determined by spectrophotometer ^24^. A reduction of cell viability superior to 30% indicates the cytotoxicity of a biomaterial ^23^. The micronucleus test evaluates the potential of the material to be toxic and to cause lesions on the genetic structure of the cell studied by means of fluorescence ^25^ ^,^ ^26^. During cellular division, in response to the damage, the cell remains viable and forms a micronucleus ^25^ ^,^ ^26^. This transformation can lead to the development of a neoplasm ^25^ ^,^ ^26^.

During the experimental periods, MTA-Ang and MTA-HP did not affect the viability of MC3T3-E1 cells, when compared to the control group. These results confirm the data of Ferreira et al. (2019), which exhibited similar rates of osteoblast viability for MTA-Ang, MTA-HP, and control ^3^. Despite BCR having caused a greater reduction in viability than MTA-Ang and MTA-HP, it was not considered cytotoxic since the viability of cells was superior to 70%. Different from other analyzed biomaterials, the ERRM was potentially cytotoxic because the viability of cells in contact with it was inferior to 70% at 3 and 7 days.

Previous studies that evaluated the cytotoxicity of ERRM and MTA-Ang ^5^ ^,^ ^11^ ^,^ ^24^ ^,^ ^5^ showed distinct results when compared with ours. Besides the cytotoxic test being the same as our study (MTT-mitochondrial tetrazolium test), the type of cells used in these studies was different from our study, influencing the results. Damas et al. ^5^ and Hirschman et al. ^11^ analyzed the biomaterials in contact with human dermal fibroblasts, differing only in the time of analysis and dilution. Araldi et al. ^26^ used human bone marrow mesenchymal stem cells, periodontal ligament stem cells, and dental pulp stem cells for 1, 3, and 7 days, and they highlighted the necessity of further studies with other parameters. Chen et al. ^27^ tested different extracts (dilutions 1:1, 1:2, 1:4, or 1:8) in contact with human periodontal ligament fibroblasts for 1, 3, and 7 days, and they observed the lower concentrations are more acceptable for cell viability. In our study, osteoblasts were used since they are present in periapical tissue and participate in repair in various clinical situations ^7^, emphasizing the importance of analysis with the type of cells.

Although ERRM and BCR do not require additional handling, some problems were encountered during sample preparation. The ERRM and BCR samples were kept in an oven at 37º C with 95% relative humidity during the setting period reported by the manufacturer, but none of them took setting in the respective period. In the cytotoxicity assay, it was observed that MTA-Ang presents an initial setting after 12 hours, while ERRM took 7 days ^5^, justifying the choice of this set time. In our study, fresh and unfiltered extracts were used conforming previously described ^28^ ^,^ ^29^. However, particles of cement were observed at the bottom of the wells which may have interfered with the obtained data. The same was observed in a previous study ^5^, and the suggested hypothesis is that although it seems that the material reaches the complete set after 7 days, it is possible that part of the material had not yet reached the ideal set internally. The particles directly in contact with the cells throughout the experimental period and not just in the extraction phase can justify the ERRM cytotoxicity potential found by the present study.

Several studies have evaluated the cytotoxicity of bioceramic cements ^3^ ^,^ ^5^ ^,^ ^6^ ^,^ ^7^ ^,^ ^11^ ^,^ ^28^, however, few studies have evaluated its DNA damage by the formation of micronuclei (Mn) ^26^. The formation of Mn was tested in fibroblasts ^9^ and hamster ovary cells ^8^ after exposure to MTA-Ang cement, and the data indicated the biomaterial induced the formation of Mn like the control group, corroborating with our results and suggesting relative security. With the evolution of bioceramics materials, one of the objectives was to preserve the biological properties of MTA, however, BCR and ERRM, which are the last-generation of bioceramics, showed genotoxicity values significantly higher than the control group, MTA-Ang, and MTA-HP. The authors suggest three hypotheses: the presence of cement particles in the wells; the packaging of these new materials; and the occurrence of a false positive result. The last hypothesis may be the result of several mechanisms, namely: primary damage to a target other than DNA, with for example damage to spindle proteins and interference with the metabolism of nucleotides and their precursors; by inducing damage by a specific process of the *in vitro* test system to DNA ^30^; by high concentrations of the product, causing detoxification or other cellular protection processes, occurring only in extreme or non-physiological conditions. However, in the face of a positive result, an evidence-based approach, evaluation of the mechanism of action, and additional support studies are necessary to deal with the result, according to The Guidance for Industry and Review Staff ^31^.

The results of this study showed that, among contemporary materials, BCR is one viable option for use as repair material in endodontics, contributing with the safety of its use in clinical practice. A previous study ^21^ has shown that BCR has similar cytocompatibility to MTA-Ang and MTA-HP; however, the present study adds that BCR also exhibits genocompatibility and is similar to ERRM. Like other last generation of cements, this is a type of reformulated material that aims to improve the properties of predecessor materials, such as ease of use, various clinical applications, and avoiding waste. In addition, it should be one more option for repairing bioceramics accessible to the public, since no pre-mixed repairing endodontic cement is available in the Brazilian market, as well as in several countries. However, it should be noted that the results of primary cytotoxicity and genotoxicity tests have some limitations and future studies are necessary for better evaluation of the material.

It can be concluded that BCR is not cytotoxic in osteoblastic cells, as well as MTA-Ang and MTA-HP. However, the genotoxicity of BCR and ERRM was increased at 7 days of treatment concerning the other groups. Although the results of this study open the door to the construction of scientific evidence for the use of new material, further investigations are needed to better elucidate the biological properties of BCR.
